# Study of Body Mass Index among Medical Students of a Medical College in Nepal: A Descriptive Cross-sectional Study

**DOI:** 10.31729/jnma.6282

**Published:** 2021-03-31

**Authors:** Reena Kumari Jha, Abuday Kumar Yadav, Sneha Shrestha, Pramit Ram Shrestha, Suyesh Shrestha, Mina Jha, Ojashwi Nepal

**Affiliations:** 1 Department of Physiology, Kathmandu University School of Medical Sciences, Dhulikhel, Kavre, Nepal; 2 Kathmandu University School of Medical Sciences, Dhulikhel, Kavre, Nepal; 3 Department of Anatomy, Janaki Medical College, Janakpur, Nepal

**Keywords:** *body mass index*, *diet*, *Nepal*

## Abstract

**Introduction:**

Changes in the lifestyle, food habits, lack of nutritious diet, stress, physical inactivity increases the body mass index among adults. Excess weight gain is an important risk factor for non-communicable diseases such as heart disease, stroke, diabetes, and some cancers (endometrial, breast, colon). Thus, this study aims to find out body mass index of medical students of a medical college in Nepal.

**Methods:**

This descriptive cross-sectional study was conducted in the department of physiology of a tertiary care center from August 2019 to February 2020 after taking ethical clearence from Institutional Review Committee (Reference number 192/19). Height and weight were recorded and body mass index was then being calculated. Data entry was done in Microsoft Excel and analyzed using Statistical Package for the Social Sciences version 22.

**Results:**

Out of 266 medical students, 39 (15%) were overweight and 32 (12%) were underweight with mean body mass index 26.60±1.99kg/m^2^ and 17.13±1.19kg/m^2^ respectively. Mean body mass index of males was 21.76±3.06kg/m^2^ and that of females was 21.70±2.96 kg/m^2^.

**Conclusions:**

Comparing with a similar study done in Nepal previously, we found a higher prevalence of overweight in medical students whereas majority of medical students had normal weight. Factors affecting body mass index in medical students should be explored further.

## INTRODUCTION

Body mass index is an index of weight for height and commonly used to classify overweight and obesity. The prevalence of overweight and obesity is increasing in low- and middle-income countries. The most important causes of obesity are increased intake of foods rich in fat, salt, sugar and lack of exercise.^1^

A linear relationship exists between body mass index and blood pressure.^2-3^ Obesity enhances sympathetic activity however blood pressure is regulated by balancing action betweentwo branches of autonomic nervous system.^4^ Cardiovascular disease in younger populations is increasing now a day due to striking shift in the lifestyle of adults. Sedentary life style as well as consumption of junk foods, andstress increases body mass index (BMI).^5^ Higher BMI increases risk for development of hypertension.^6^

The aim of this study was to find out the mean body mass index of medical students and measure their dietary habits.

## METHODS

This descriptive cross-sectional study was carried among undergraduates of Kathmandu University School of Medical sciences, Chaukot, Kavre from August 2019 to February 2020 after taking ethical clearence from Institutional Review committee of Kathmandu University School of Medical Sciences/Dhulikhel Hospital (IRC-KUSMS192/19). Subjects suffering with cardiorespiratory disease and autonomic dysfunction were excluded from the study. Sample size can be calculated by following formula.

$$\begin{array}{ll} \text{n} & =({\text{z}}^{\text{2}}\times {\text{σ}}^{\text{2}}\text{)}/{\text{e}}^{\text{2}}\\ & =({\left(1.96\right)}^{2}\times {\left(0.5\right)}^{2})/{\left(0.06\right)}^{\text{2}}\\ & =267 \end{array}$$

Where,

Z = 1.96 at 95% Confidence Interval
a = Standard deviation, 0.5
e = margin of error, 0.06

Adjusting the sample size for finite population,

$$\begin{array}{ll} \text{n} & ={\text{n}}_{\circ }/[1+\{(n_{\circ }-1)/N\}]\\ & =267/[1+\{267-1)/865]\\ & =207 \end{array}$$

where,

n_∘_ = required sample size
n = adjusted sample size
N = Total students in MBBS, BDS, BPT, and BNS faculties, 865

The required sample size was taken as 266. Therefore, we included 266 participants in the study using convenience sampling technique. First the subject was informed about the procedure and the consent was taken. Then the subject was asked to fill up the questionnaire that includes detailed history regarding the dietary habits and history suggestive of any cardio-respiratory or any other systemic illness. Based on the information obtained from the questionnaire, the participants were divided into two groups such as vegetarians and non-vegetarians. Those who consumed foods of plant and animal source including meat, fowl, eggs, and fish were considered as nonvegetarian and those who consumed food from only plant source and milk were classified as vegetarians.

Height was measured without shoes, to the nearest 0.5cm with participant standing erect against the wall with heels together and touching the wall, and head held in upright position. Weight was measured with minimum cloths and no footwear on a standardized weighing machine marked from 0 to 130 kg and was recorded to the nearest 0.5 kg. Body mass index (BMI) was then calculated using the formula weight in kilograms divided by the square of the height in meters{weight (kg)/ height (m^2^)}. It was then summarized and categorized into three groups, underweight (BMI <18.5kg/m^2^), normal weight (BMI 18.5 to 24.9kg/m^2^), overweight (BMI 25.0-29.9kg/m^2^) and obese (BMI 30 - 34.9kg/m^2^) in accordance with the WHO recommendation.^7^

Data were entered into Microsoft Excel and analyzed using IBM Statistical Package for the Social Sciences version 22 software. The data were analyzed using descriptive statistics and have been presented as means, standard deviations, frequencies, and percentages.

## RESULTS

Out of the total medical students in this study, the mean body mass index of all participants was 21.70±1.96. Of them, 130 (49%) males and 136 (51%) females, age ranging from 17-25 years were included in this study. Thirty-nine students (14.66%) were overweight with mean body mass index of 26.60±1.99kg/m^2^. One hundred ninety-five (73.30%) students had normal body mass index and some 32 (12.3%) students were underweight (Table 1).

**Table 1:** Demography data of medical students.

Groups	n (%)	BMI (kg/m^2^) (Mean±SD)
Underweight (BMI^*^<18.5)	32 (12.03)	17.13±1.19
Normal weight (BMI 18.5 - 24.9)	195 (73.30)	21.46±1.64
Overweight (BMI>25)	39 (14.66)	26.60±1.99

* BMI - Body Mass Index

Height, weight and body mass index of male subjects were higher than female subjects (Table 2).

**Table 2:** BMI of male and female medical students.

Parameters	Male (Mean±SD)	Females (Mean±SD)
Height (m)	1.7±0.06	1.58±0.058
Weight (kg)	62.89±9.29	54.10±8.74
BMI^*^	21.76±3.06	21.70±2.96

* BMI - Body Mass Index

Out of 266 participants, 34 (12.78%) vegetarians with mean body mass index of 20.72±2.95kg/m^2^ and 232 (87.21%) non-vegetarians with mean BMI of 21.84±2.94kg/m^2^ (Table 3).

**Table 3:** Diet of the participants.

	n (%)	BMI^*^(kg/m^2^) (Mean ± SD)
Vegetarian	34 (12.78)	20.72±2.95
Nonvegetarian	232 (87.21)	21.84±2.94

* BMI - Body Mass Index

## DISCUSSION

This was a cross-sectional descriptive study based on self structured questionnaire, where we assessed BMI, dietary habits, blood pressure and heart rate of medical students. The prevalence of obesity is increasing continuously worldwide, affecting all ages, sexes, races and becoming major risk factor for non-communicable disease.^1^ We found in our study that 14.66% medical students were overweight. Similar studies that calculated BMI of medical students have been conducted in India, Pakistan, Poland, United Arab Emirates and Greece.^8-11^ Amatya et al.^12^ in their study found that 18.31% medical students underweight, 77.18% normal, 4.23% overweight, and 0.3% obese (one male student).

Our study showed that the mean height, weight and BMI of the male participants were found to be higher than their female counterparts. Similar results were found by previous study.^13,14^ The difference in weight could be attributed to the difference in bone density, where the bones of males are denser than that of females. The authors found that there is positive association between BMI and height during prepubertal children.^13^ In contrast to our study, researcher found that BMI is negatively related with height particularly in young women.^15^

In the present study, body mass index were found to be higher in nonvegetarian than vegetarians. This is consistent with previous study conducted by several researchers.^16-18^ Comparison of vegetarian and nonvegetarian diet shows that vegetarian diets are usually rich in carbohydrates, dietary fiber, carotenoids, folic acid, vitamin C, vitamin E, magnesium and relatively low in protein, saturated fat, retinol, vitamin B12, Zinc.^19^ Higher intake of dietary fiber and lower intake of animal fat can reduce body mass index.^16^ The extensive reviews of observational studies that used eating pattern methods suggest that fiber rich foods, such as vegetables, fruits, cereals, whole grains, and legumes, is inversely related to body mass index (BMI), overweight, and obesity.^18^

We conducted this study in a limited sample size in a single institution. Our study design didn't permit the measurement of association between variables. Further studies done multiple institutes with a larger sample size must be carried out to find out the true prevalence and mean values of body mass indices of Nepalese medical students.

## CONCLUSIONS

Comparing to findings of previous research done in Nepal, we found higher prevalence of overweight in medical students. Male had higher overweight than female. Among nonvegetarians body mass index was found to be higher. Regular physical exercise and balanced diet should be followed to prevent overweight and obesity.
